# Edge Detection of Motion-Blurred Images Aided by Inertial Sensors

**DOI:** 10.3390/s23167187

**Published:** 2023-08-15

**Authors:** Luo Tian, Kepeng Qiu, Yufeng Zhao, Peng Wang

**Affiliations:** 1 Department of Precision Instrument, Tsinghua University, Beijing 100084, China; tian-l18@mails.tsinghua.edu.cn (L.T.); qiukp@mail.tsinghua.edu.cn (K.Q.); 2 Heilongjiang North Tool Co., Ltd., Mudanjiang 157000, China; zhaoyufeng@beifang.ntesmail.com

**Keywords:** edge detection, image deblurring, inertial sensor, neural network

## Abstract

Edge detection serves as the foundation for advanced image processing tasks. The accuracy of edge detection is significantly reduced when applied to motion-blurred images. In this paper, we propose an effective deblurring method adapted to the edge detection task, utilizing inertial sensors to aid in the deblurring process. To account for measurement errors of the inertial sensors, we transform them into blur kernel errors and apply a total-least-squares (TLS) based iterative optimization scheme to handle the image deblurring problem involving blur kernel errors, whose relating priors are learned by neural networks. We apply the Canny edge detection algorithm to each intermediate output of the iterative process and use all the edge detection results to calculate the network’s total loss function, enabling a closer coupling between the edge detection task and the deblurring iterative process. Based on the BSDS500 edge detection dataset and an independent inertial sensor dataset, we have constructed a synthetic dataset for training and evaluating the network. Results on the synthetic dataset indicate that, compared to existing representative deblurring methods, our proposed approach demonstrates higher accuracy and robustness in edge detection of motion-blurred images.

## 1. Introduction

To initiate an exploration into the realm of image processing, one must be drawn to the significance of edges—the fundamental features that underpin visual information. In practical applications, edge detection serves as a pivotal low-level operation, forming the bedrock for various high-level tasks such as feature extraction [1], image segmentation [2], object recognition [3], and object proposal [4]. However, when there is relative motion between the camera and the object during the exposure time, the captured image will appear with motion blur. The direct application of edge detection on motion-blurred images leads to significantly reduced accuracy due to the presence of artifacts, thereby affecting subsequent image processing tasks. Currently, there are two main challenges in performing edge detection on motion-blurred images.

Firstly, edge detection of motion-blurred images requires an effective deblurring method. In previous work, the majority of efforts have focused on utilizing the content of the image itself to remove motion blur [5,6,7,8], and it is an ill-posed problem since both the latent image and the blur kernel remain unknown. Inertial sensors, such as gyroscopes and accelerometers, can provide additional motion information about the imaging system during exposure. Utilizing inertial sensors to assist in deblurring can effectively reduce the ill-posedness of deblurring algorithms [9]. Nevertheless, due to time synchronization error and noise, accurate motion information cannot be obtained from sensor data, thus the deblurring methods aided by inertial sensor data often lack robustness [10].

On the other hand, motion deblurring methods have been developed for improvement of the image quality but are not designed for a better edge structure perception. Recent studies have demonstrated that compared to deblurring results with lower peak signal-to-noise ratio (PSNR), deblurring results with higher PSNR do not always achieve better performance in edge detection [11]. Coupling deblurring algorithms with edge detection tasks and ensuring that the deblurring method can effectively improve the edge detection accuracy is another key problem to be addressed.

Based on the above issues, our work made the following contributions:

- The sensor data with errors are transformed into blur kernel with errors, and we apply a TLS-based iterative optimization scheme to handle the image deblurring problem involving blur kernel errors, whose relating priors are learned by two types of neural networks. The inclusion of the blur kernel with sensor data error information in the training process makes the final deblurring method strongly robust.
- The canny edge detection algorithm is incorporated into the deblurring process for calculation of the final loss function. By coupling the edge detection task and the deblurring iterative process more tightly, we ensure that the edge detection task achieves higher accuracy through the image deblurring process.
- The BSDS500 edge detection dataset and an independent inertial sensor dataset are combined to create a synthetic dataset for edge detection of motion-blurred images. The results for the synthetic dataset demonstrates the effectiveness and robustness of the proposed method.

## 2. Related Work

Deblurring methods have remained an active research area in recent years and usually requires priori knowledge or additional capture information to obtain a valid solution [12,13]. More accurate information about the camera motion can be obtained using inertial sensors, such as gyroscopes and accelerometers, which have been successfully utilized to assist in motion deblurring. Joshi et al. built a single lens reflex camera equipped with a gyroscope and accelerometer to estimate the motion of the camera over the course of the exposure [9]. The sensor data was corrected beforehand under the guidance of a natural image. Park and Levoy employed a similar gyroscope calibration method to address the multiple image deblurring problem [14]. Šindelář and Šroubek developed a real-time deblurring method on mobile devices based on a spatially invariant blur approximation [15]. Due to hardware precision limitations, the sensor data play only a qualitative role in the blur kernel estimation. Zhang and Hirakawa combined inertial measurements and image based information to remove the blur [16]. All of these works assume that the sensor data they recorded is reliable, or they only slightly relax this assumption. However, there are more challenging problems when using inertial sensors that do not provide high quality sensor data for effective image clarification.

Mustaniemi et al. applied gyroscopic data for the first time for single image deblurring based on deep learning networks [17]. In their work, sensor data errors have been taken into account during network training. However, they only used the sensor data to simplify the shape of the blur kernel to a straight line, which would result in partial loss of information in the physical phase and thus would have an impact on the final performance of the Convolutional Neural Network (CNN). Nan and Ji recently proposed a TLS-based iterative optimization scheme for dealing with the kernel error problem in image deblurring [18], which can obtain good deblurring results even when there is an error in the estimation of the blur kernel. This framework is particularly suitable for handling blur kernel errors caused by sensor data errors.

In the aspect of coupling deblurring algorithms with edge detection tasks, an extensive theoretical overview of task-adapted image reconstruction was presented in the work by Adler et al. [19]. Their study revealed that joint reconstruction-segmentation approaches achieved more accurate segmentations compared to both sequential and end-to-end methods. Yang et al. proposed a new cooperative game framework for joint image restoration and edge detection [20]. It used an iterative approach to solve the two tasks, and the interactive facilitation between the tasks during iteration resulted in improvements in both image restoration and edge detection performance.

Despite these efforts, effectively addressing edge detection tasks in the presence of motion blur remains a challenging endeavor. The key aspect lies in ensuring the efficacy of the deblurring process while also striving to achieve higher edge detection precision with the deblurred results.

## 3. Method

### 3.1. Initial Kernel Estimation

Recall the spatially-invariant convolutional model of the image blurring process: g=f∗k+n, where g and f denote the motion-blurred image and its latent sharp image, * is the convolution operator, k is the blur kernel and the noise term n is often formulated as the additional white Gaussian noise.

In order to incorporate the inertial sensor data that record the camera motion information, the motion-blurred image is formulated as the summation of multiple sharp images under a sequence of projective motions during the exposure interval:

(1) $$g\left(x\right)=\frac{1}{Np}\sum_{t=1}^{N_{p}}f\left(H_{t}x\right)+n$$

where $x\in {\mathbb{R}}^{3\times 1}$ denotes the pixel location in homogeneous coordinate, $H_{t}$ denotes the homography matrix, n denotes the noise term and $N_{p}$ denotes the number of all camera poses during the exposure time. Considering the planar homography that maps the initial projection of points at t=0 to any other time t, the homography matrix $H_{t}$ can be characterized as [21]:

(2) $$H_{t}=K(R_{t}+\frac{T_{t}N^{T}}{d})K^{-1}$$

for a particular depth *d*. $R_{t}$ is the rotation transformation matrix, $T_{t}$ is the translation vector, and $N^{T}$ is the unit vector that is orthogonal to the image plane. The camera intrinsic matrix K can be characterized by the focal length f and camera optical center ($O_{x}$, $O_{y}$):

(3) $$K=\left[\begin{array}{ccc} f & 0 & O_{x}\\ 0 & f & O_{y}\\ 0 & 0 & 1 \end{array}\right]\;$$

**Figure 1:**
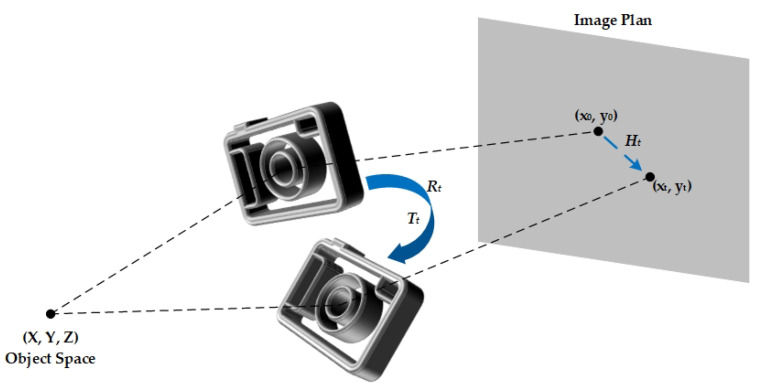
Image blurring model caused by camera motion.

Parameters related to motion, the rotation transformation matrix $R_{t}$ and the translation vector $T_{t}$, can be calculated from the measurements of the gyroscope and accelerometer, respectively. The process of image blurring caused by camera motion is shown in Figure 1.

Assuming that the rotation center locates at the optical center of the camera, the rotation transformation matrix can be approximated as

(4) $$R_{t}=\left[\begin{array}{ccc} 1 & -d\theta_{t}^{z} & d\theta_{t}^{y}\\ d\theta_{t}^{z} & 1 & -d\theta_{t}^{x}\\ -d\theta_{t}^{y} & -d\theta_{t}^{x} & 1 \end{array}\right]R_{t-1}$$

by employing the sinusoidal approximation when the angular rotation is small. Given the gyroscope measurement $\omega_{t}={\left[\omega_{tx},\;\omega_{ty},\;\omega_{tz}\right]}^{T}$ at time *t* and the sampling interval ∆t, ${\left[d\theta_{t}^{x},\;d\theta_{t}^{y},\;d\theta_{t}^{z}\right]}^{T}=\omega_{t}\times ∆t$. Since only relative rotation is considered, the initial rotation transformation matrix $R_{0}=Identity$.

As for the translation vector $T_{t}$, since there are currently mobile devices that can eliminate the influence of gravity using data from other sensors, we can perform a double integration on the accelerometer measurement $a_{t}={\left[a_{tx},\;a_{ty},\;a_{tz}\right]}^{T}$ without subtracting the gravitational acceleration.

After calculating the homography matrix $H_{t}$ at any given moment *t*, we can ultimately obtain the projected trajectory within the exposure time. Moreover, we approximate the blur kernel of the entire image using the projected trajectory of the central pixel point. Specifically, we set all pixel values to 0 except for the central coordinates, where the pixel value is set to 255, to obtain the image $f^{c}\left(x\right)$. Then, we set the white noise to 0 and use $f^{c}\left(x\right)$ as input in Equation (1) to compute the image $g^{c}\left(x\right)$, which represents the initial blur kernel k.

Due to the influence of time synchronization error and noise, the direct calculation of blur kernel using sensor data will lead to the difference between the estimated blur kernel and the exact blur kernel. Thus, the problem turns to how to use the blur kernel with errors for image deblurring.

### 3.2. TLS-Based Iterative Optimization Scheme for Blur Kernel with Errors

When considering the blur kernel with errors, the image blurring model is as follows

(5) $$g=\left(\hat{K}-\Delta K\right)f+n=\hat{K}f-\Delta Kf+n\;$$

where $\hat{K}$ is the matrix form of the convolution operator.

The TLS estimator finds the solution to (5) through the resolution of a constrained optimization problem.

(6) $$\underset{\Delta K,n,f}{min}{\left\|\Delta K\right\|}_{F}^{2}+{\left\|n\right\|}_{2}^{2}\;\;\;s.t.\;\left(\hat{K}f-\Delta Kf=g-n\right)\;$$

By introduction of an auxiliary variable ***u*** that represents the kernel error term ΔKf, we reformulate the problem (6) into an optimization problem as follows:

(7) $$\underset{f,u}{min}{\left\|\Delta K\right\|}_{F}^{2}+{\left\|g-\hat{k}*f-u\right\|}_{2}^{2}+\lambda {\left\|u-\Delta Kf\right\|}^{2}+\Phi \left(f\right)\;$$

where $\Phi \left(f\right)$ denotes the regularization term with respect to certain image prior which is usually imposed on high-frequency image components, as they are the main parts lost in the blurring process. By introduction of an auxiliary variable z and applying the half-quadratic splitting, the problem (7) can be reformulated as:

(8) $$\underset{f,u,z}{min}{\left\|g-\hat{k}*f-u\right\|}_{2}^{2}+\varphi (u|f)+{\left\|diag\left(\lambda \right)\left(\Gamma f-z\right)\right\|}_{2}^{2}+\rho \left(z\right)$$

where $\varphi \left(u|f\right)=\underset{\Delta K}{min}{\left\|\Delta K\right\|}_{F}^{2}+\lambda {\left\|u-\Delta Kf\right\|}^{2}$ is the regularization term related to the prior imposition on the correction term caused by kernel error and Γ denotes high-pass filters. An alternating iterative scheme can be employed to solve the optimization problem (7):

(9) $$f^{\left(t\right)}=\underset{f}{argmin}{\left\|g-\hat{k}*f-u^{\left(t-1\right)}\right\|}_{2}^{2}+\lambda {\left\|diag\left(\lambda \right)\left(\Gamma f-z^{\left(t-1\right)}\right)\right\|}_{2}^{2}\;$$

(10) $$z^{\left(t\right)}=\underset{z}{argmin}\mu {\left\|\Gamma f^{\left(t\right)}-z\right\|}_{2}^{2}+\rho \left(z\right)$$

(11) $$u^{\left(t\right)}=\underset{u}{argmin}{\left\|g-\hat{k}*f^{\left(t\right)}-u\right\|}_{2}^{2}+\varphi (u|f^{\left(t\right)})$$

The first step (9) is an inversion process which can be solved using discrete Fourier transform given $u^{\left(t-1\right)}$ and $z^{\left(t-1\right)}$ of last iteration.

The second step (10) is a denoising process, which eliminates potential artifacts present in the high-pass image channels. The CNN-based denoising neural network called Dn-CNN [22] can be used to remove noise in $f^{\left(t\right)}$.

The third step (11) is a correction process, which corrects the term relating to kernel error. Proposed by Nan and Ji [18], the neural network Dual-Path U-net can be used as a tool to estimate the correction term $u^{\left(t\right)}$ by combining the downsampled codes from $f^{\left(t\right)}$ and the residual $g-\hat{k}*f^{\left(t\right)}$.

By adopting the above framework, we have obtained an effective deblurring method assisted by inertial sensors which could handle blur kernel errors caused by sensor data errors. Our proposed deblurring method can be described as Algorithm 1.

**Algorithm 1:** Deblurring Assisted by Inertial Sensors**Input:** gyroscope data ${\left\{w\right\}}_{i}$, accelerometer data ${\left\{a\right\}}_{i}$, blurred image ***g*** **Output:** deblurred image ***f*** **Procedure:**
(1)obtain ${\left\{T\right\}}_{t}$ performing a double integration on ${\left\{a\right\}}_{i}$(2)obtain ${\left\{R\right\}}_{t}$ using (4)(3)obtain ${\left\{H\right\}}_{t}$ using (2), (3)(4)set the center pixel to 255, and all other pixels to 0, obtaining the image $f^{c}$(5)obtain blur kernel ***k*** applying (1), using all the obtained ${\left\{H\right\}}_{t}$ and the image $f^{c}$ with the noise set to 0(6)initialize ***z***^0^ and ***u***^0^ to 0(7)obtain ***f***^0^ using discrete Fourier transform to solve (8) with blur kernel ***k***(8)**for** *iter* = 1 to *N* **do**
  obtain ***z^iter^*** using Dn-CNN with ***f***^***iter***−1^  obtain ***u^iter^*** using DP-Unet with blur kernel ***k*** and ***f***^***iter***−1^  obtain ***f^iter^*** using discrete Fourier transform to solve (8) with blur kernel ***k***, ***z^iter^*** and ***u^iter^***
**end**(9)***f^N^*** is the final output ***f***
**end**


### 3.3. Overall Network Structure with Canny Edge Detection Algorithm Added

**Figure 2:**
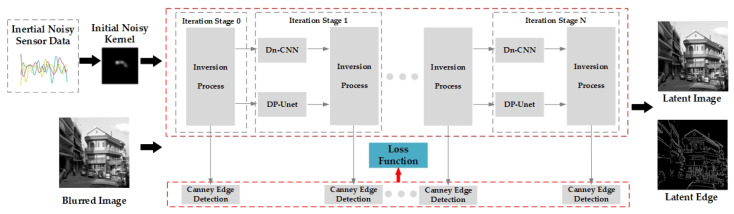
Diagram of the proposed deblurring method.

The loss function in the framework proposed by Nan and Ji is defined as [18]:

(12) $$L=\frac{1}{J}\overset{J}{\underset{j=0}{\sum }}\left({\left\|f_{j}^{\left(T,+,1\right)}-f_{j}\right\|}_{2}^{2}+\overset{T}{\underset{i=2}{\sum }}\mu_{i}{\left\|f_{j}^{\left(i\right)}-f_{j}\right\|}_{2}^{2}\right)\;$$

where $\left\{f^{\left(1\right)},\;f^{\left(2\right)},\;\ldots ,\;f^{\left(T+1\right)}\right\}$ are the sequence of deconvoluted images corresponding to T+1 iterations in the optimization algorithm. To ensure that the network’s optimization goal is to improve the accuracy of edge detection, we incorporate the effect of edge detection results into the iterative process. Specifically, at each step of the iterative process, we perform Canny edge detection [23] on the output of Equation (11) and use the edge detection result to calculate the edge cross-entropy function

(13) $$l_{edge}^{\left(i\right)}=-\frac{1}{J}\overset{J}{\underset{j=0}{\sum }}\left(e_{j}^{\left(i\right)}\log(\hat{e_{j}^{\left(i\right)}})+\left(1-e_{j}^{\left(i\right)}\right)\log(1-\hat{e_{j}^{\left(i\right)}})\right)$$

where $e_{j}^{\left(i\right)}$ is the edge detection result of $f^{\left(i\right)}$ and $\hat{e_{j}^{\left(i\right)}}$ represents the edge ground truth. The overall loss function is redefined as

(14) $$L=l_{edge}^{\left(T+1\right)}+\overset{T}{\underset{i=2}{\sum }}\mu_{i}l_{edge}^{\left(i\right)}\;$$

where the weights ${\left\{\mu_{i}\right\}}_{i=1}^{T-1}$ are set to 0.8. In summary, our deblurring method can be represented by the schematic diagram in Figure 2.

Incorporating edge detection into the deblurring process and using the edge loss function to adjust network parameters can encourage the model’s output to be close to the ground truth edges, as specified by the first term in Equation (14). This can be achieved using currently advanced deblurring methods. However, our approach differs in that we use an iterative optimization-based deblurring algorithm, which allows us to obtain intermediate edge detection results. This ensures that the intermediate results are not too far from the ground truth edges, as specified by the second term in Equation (14). Indeed, our method incorporates richer edge information and is expected to perform better in edge detection tasks for motion-blurred images.

### 3.4. Synthetic Dataset of Motion-Blurred Images with Inertial Sensor Data

Sufficient high-quality training samples are essential for deep learning-based models. We propose a method for synthesizing a comprehensive dataset that includes ground truth edges, blurred images, and inertial sensor data captured during the exposure time of each blurred image.

We construct our dataset based on the real images and their corresponding ground truth edges from the BSDS500 dataset [24]. The BSDS500 dataset has already partitioned the data into training, validation, and test sets. For each image and its ground truth in the BSDS500 training set, we crop them into patches of 256 × 256. In the direction with pixel value 321, the first cropping uses a stride of 1 pixel, and subsequently, a step size of 32 pixels is used, resulting in a total of 4 patches. In the direction with pixel value 481, the first three croppings use a stride of 1 pixel, the fourth cropping uses a stride of 2 pixels, and thereafter, a stride of 4 pixels is used, resulting in a total of 60 patches (The purpose of doing this is to make the number of patches obtained from cropping adaptable to a wider range of batch sizes). As a result, 200 × 4 × 60 = 48,000 sharp images along with their corresponding ground truth edges are obtained.

**Figure 3:**
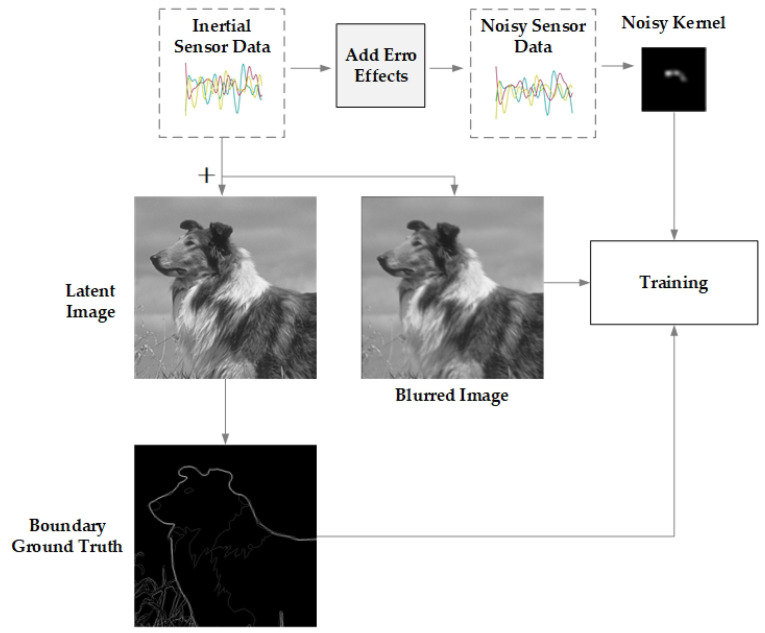
Synthetic training data generation scheme.

The inertial sensor data, specifically the angular velocity and the acceleration of each axis, are modeled using a Gaussian distribution with a mean of 0. The standard deviation of angular velocity of each axis is $\sigma_{\omega x}=\sigma_{\omega y}=1$ × 10^−6^ rad/s and $\sigma_{\omega z}=0.1$ rad/s; the standard deviation of acceleration of each axis is $\sigma_{ax}=\sigma_{ay}=1$ × 10^−3^ m^2^/s and $\sigma_{\omega z}=1$ × 10^−5^ m^2^/s. After randomly determining the exposure time within the range of (0.02, 0.2) seconds, we sample the sensor data within the exposure time at a frequency of $f_{s}=200\;\mathrm{Hz}$. To simulate continuous motion, each sensor data sample is interpolated from the preceding data point, with angular velocity data linearly and acceleration data approximated. Utilizing the sharp images and their edges ground truth obtained from cropping the BSDS500 training set, combined with the generated sensor data, the overview of our training data generation scheme is shown in Figure 3.

It is essential to note that the exact sensor data is directly combined with the clear images to calculate the motion-blurred images for the training set, while the sensor data with errors, obtained by adding synchronization errors and noise terms to the exact sensor data, is used to compute the estimated blur kernel for the training set. Following the steps outlined in [25], we set the time delay $t_{d}$ randomly picked from a Gaussian distribution *N* $\left(0.03,\;0.01^{2}\right)$ in seconds, and the noise for inertial sensor data as additive white Gaussian noise with standard deviation as 1/10 of the standard deviation of corresponding data.

The validation and test set images of the BSDS500 dataset are not cropped, and we also employ the aforementioned scheme to generate the validation and test sets for our dataset.

## 4. Experiments

### 4.1. Experimental Setup

**Figure 4:**
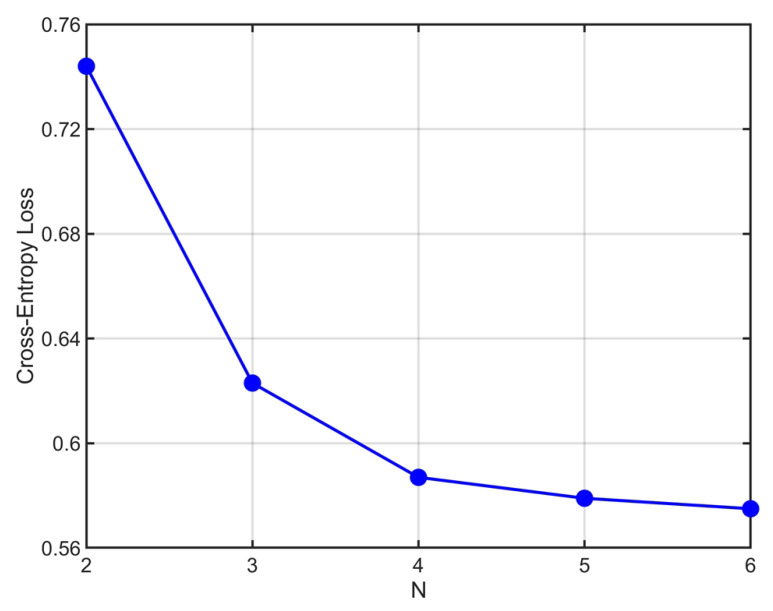
Cross-entropy loss of the final iteration by varying the iteration parameter N.

As for $\left\{\lambda^{\left(t\right)}\right\}$ in Equation (9), we set $\lambda^{\left(0\right)}=0.005$ for stage 0 and $\lambda^{\left(t\right)}=0.5$ for later stage. The network is trained using the Adam optimizer [26]. The learning rate, training batch size and the number of epochs for network training are set to be 1 × 10^−4^, 4 and 100, respectively. The iterative parameter N has a significant impact on the performance of the approach we proposed. Therefore, we determine its value through the heuristic method. We set the value of N to range from 2 to 6 and exploit the cross-entropy loss of the final iteration with different N. Figure 4 shows the loss trend on the test set with the change of N. It can be observed that the performance improvement becomes marginal when N exceeds 4. Considering that the network architecture should not be overly complex, N is set to 4.

Since there may be random dislocation between the deblurred image and the corresponding sharp image, we adopt the same procedure as described in [27] to align the deblurred images with the sharp images and then cut off the boundary pixels. Before evaluating the results on the test set, the same alignment operation is performed on the edge detection results, ensuring they are accurately aligned with the ground truth edges.

### 4.2. Ablation Study

Our ablation study focuses on the performance gain brought by the introduction of intermediate edge detection results in the iterative optimization process. We consider the following three cases for comparison: the original deblurring network structure without edge information, incorporating edge information only in the final output and incorporating richer intermediate edge information during the iterative process(ours). We keep the training settings consistent, and the same Canny edge detection algorithm is applied to obtain the edge detection results for all the deblurred results. The edge detection performance on the test set of our proposed synthetic dataset is shown in Table 1, and the F-measure at both Optimal Dataset Scale (ODS) and Optimal Image Scale (OIS) are recorded for evaluation. The F-measure is a widely used metric in edge detection evaluation, and it balances the precision and recall of detected edges. ODS refers to computing the F-measure by selecting the best threshold for each individual image in the dataset, while OIS calculates the F-measure by choosing the optimal threshold globally across all images. These metrics are crucial for assessing the performance of our proposed approach for edge detection in motion-blurred images.

It can be seen from Table 1 that our method achieves the highest ODS and OIS scores while the original deblurring network structure without edge information performs the worst, which indicates that incorporating richer edge information during the deblurring process can indeed improve the edge detection performance.

**Figure 5:**
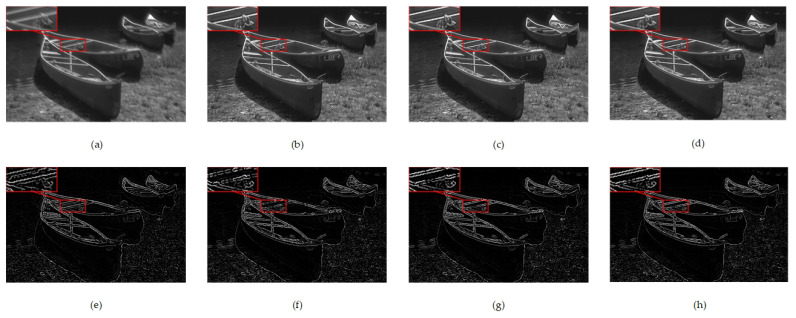
Visual inspection of ablation study. (**a**,**e**) Motion-blurred image and its edge detection result; (**b**,**f**) Deblurred result of the network without edge information and its edge detection result; (**c**,**g**) Deblurred result of the network with output edge information and its edge detection result; (**d**,**h**) Deblurred result of our network with intermediate edge information and its edge detection result.

See Figure 5 for the comparison of the deblurred and edge detection results for these three methods. Figure 5a shows a motion-blurred image, while Figure 5e displays its edge detection result. Figure 5b–d depict the deblurred results of these three methods: the network without edge information, the network with output edge information, and our proposed network with richer intermediate edge information, and Figure 5f–h represent the edge detection results obtained after deblurring the images using these three methods. Due to the incorporation of richer edge information during training, our proposed method enhances the contrast between objects and the background when deblurring motion-blurred images. Although this may increase the gap between the deblurred result and the sharp image, it improves edge detection performance and produces clearer and more stable edges.

### 4.3. Performance Evaluation and Comparison

The proposed method is compared with representative image deblurring methods. The competing methods include traditional single image-based deblurring method hyper-Laplacian (HL) [28] and deep learning-based deblurring methods DeblurGAN-v2 [29], FDN [30] and IRCNN [31], and the same Canny edge detection algorithm is applied to obtain the edge detection results for all the deblurred results. DeblurGAN-v2 [29] is a blind deblurring method, while FDN [30] and IRCNN [31] are non-blind deblurring method swhere the blur kernels are estimated using the method described in Section 3.1, and all these deep learning-based methods have been retrained on our synthetic dataset to establish fair play.

**Figure 6:**
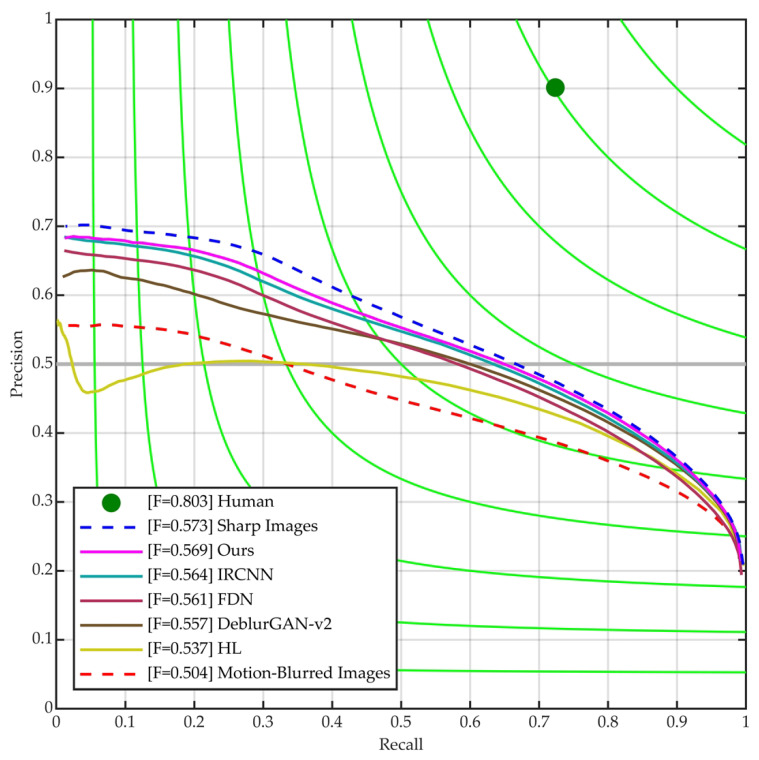
Precision-Recall curves of our method and some competitors on synthetic dataset.

Figure 6 and Table 2 show the edge detection results of all the methods. It can be seen that the ODS and OIS scores of our proposed method is modestly better than FDN [30] and IRCNN [31] and surpasses HL [28] and DeblurGAN-v2 [29] by a significant margin. This indicates that our method outperforms these existing methods in terms of edge detection performance for motion-blurred images.

**Figure 7:**
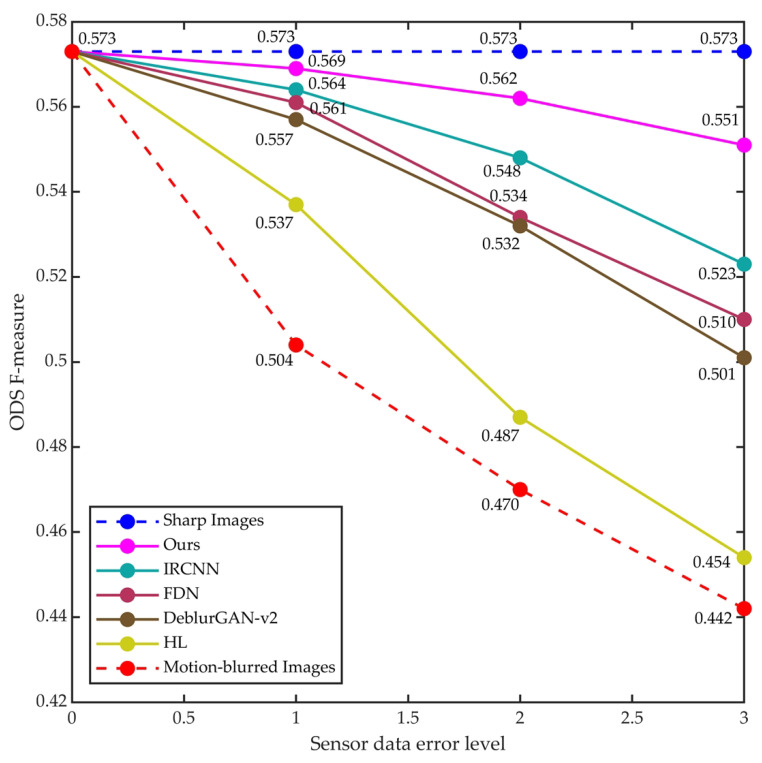
Robustness test results of our method and other methods on synthetic dataset.

To test the robustness of the proposed method against sensor data errors, we amplified the sensor data error levels in the synthetic dataset by a factor of 2 and 3 (the exposure time was also scaled by a factor of 2 and 3 correspondingly) to generate new test images for evaluating the edge detection performance of the above methods. The comparison results with other methods are shown in Figure 7. It can be clearly seen that the accuracy of our proposed method decreases less as the error level increases, which indicates the robustness of our method in dealing with sensor data errors.

## 5. Conclusions

In this paper, we propose an approach that ensures the efficacy of the deblurring process while coupling it with the edge detection task, thereby achieving higher edge detection precision. We utilize inertial sensors to aid in the deblurring process and address the impact of sensor data errors through a NN-based iterative optimization scheme. During the iterative process, we incorporate rich edge information to adapt the network’s optimization objective to edge detection tasks. Experimental results show that our proposed method achieves higher accuracy and robustness on a synthetic dataset, demonstrating the effectiveness of our method for edge detection of motion-blurred images.

In our future work, we are committed to advancing our research by constructing an image acquisition platform that incorporates inertial sensor data. By capturing real-world motion-blurred image data along with inertial sensor data during exposure time, we will compare the results of deblurring and edge detection with other methods in terms of size measurement accuracy. This evaluation will allow us to better validate and showcase the efficacy of our algorithm in practical scenarios and enable comprehensive comparisons with currently advanced deblurring methods.

## Figures and Tables

**Table 1:** Edge detection performance of ablation study on synthetic dataset.

Method	ODS	OIS
w/o edge info	0.558	0.585
with output edge info	0.566	0.593
ours	**0.569**	**0.596**

**Table 2:** Edge detection performance of our method and some competitors on synthetic dataset.

Method	ODS	OIS
Sharp Images	0.573	0.605
Motion-Blurred Images	0.504	0.540
HL [28]	0.537	0.576
DeblurGAN-v2 [29]	0.557	0.583
FDN [30]	0.561	0.581
IRCNN [31]	0.564	0.590
Ours	**0.569**	**0.596**

## Data Availability

Not applicable.
